# SIPdb: a stable isotope probing database and analytical dashboard for linking amplicon sequences to microbial activity using reverse ecology

**DOI:** 10.1093/ismeco/ycag216

**Published:** 2026-07-28

**Authors:** Alex Batista Trentin, Abigayle Simpson, Jeffrey A Kimbrel, Steven J Blazewicz, Roland C Wilhelm

**Affiliations:** Agronomy Department, Lilly Hall of Life Sciences, Purdue University, West Lafayette, IN 47907, United States; Agronomy Department, Lilly Hall of Life Sciences, Purdue University, West Lafayette, IN 47907, United States; Department of Ecology and Evolutionary Biology, Steinhaus Hall, University of California, Irvine, CA 92697, United States; Physical and Life Sciences Directorate, Lawrence Livermore National Laboratory, Livermore, CA 94550, United States; Physical and Life Sciences Directorate, Lawrence Livermore National Laboratory, Livermore, CA 94550, United States; Agronomy Department, Lilly Hall of Life Sciences, Purdue University, West Lafayette, IN 47907, United States

**Keywords:** stable isotope probing, reverse ecology, isotope incorporators, ecological inference gap

## Abstract

Stable isotope probing (SIP) connects microbial sequence data to diverse metabolic activities, but the lack of a unifying framework for SIP-derived data has limited its integration into broader strategies for ecological inference. Here, we introduce the SIPdb, an extensible SQLite database of curated nucleic acid SIP experiments (also in phyloseq format) paired with an interactive RShiny dashboard for analysis and visualization. The initial release compiles 22 studies covering 21 isotopolog substrates across diverse environments, standardized using the MISIP metadata standard. SIPdb provides a standardized pipeline accommodating the three most common SIP gradient fractionation strategies (binary, multi-fraction, and density-resolved), two incorporator designation strategies (fixed- and sliding-window), and four complementary differential abundance methods (DESeq2, edgeR, limma-voom, and ALDEx2). Using this pipeline, we identified over 42 000 unique amplicon sequence variants as isotope incorporators across 62 phyla. Benchmarking with SIPSim-generated synthetic datasets showed that position-resolved designs performed best and that differential abundance method contributed comparably to variation in incorporator designation, with DESeq2 suggested as a balanced default. Validation against original publications showed that, on average, SIPdb recovered 70.1% of author-reported incorporators, with discrepancies arising from differences in phylotyping or classification approaches. Finally, reanalysis of a non-SIP study of 1,4-dioxane degradation showed how SIPdb can both validate known degraders and uncover additional candidate taxa involved in community metabolism. SIPdb establishes a scalable platform for reverse ecology, enabling hypothesis generation, cross-study meta-analysis, and linking taxa to metabolic processes, while serving as an open, extensible resource to accelerate ecological interpretation in microbiome research.

## Introduction

Although the DNA sequencing revolution has significantly advanced our understanding of microbial diversity, efforts to interpret the ecological roles of community members remain limited by a persistent gap between sequence-based surveys and verified ecological function, which we refer to here as the ecological inference gap [1, 2]. This gap reflects a fundamental mismatch between commonly used sequencing methodologies and the inherent ecological and evolutionary properties of microbial systems, including variable gene copy numbers, strain-level heterogeneity, and the sparse availability of directly observed functions and activities [3–6]. A major contributor to this gap is the reliance on culture-based characterizations as a proxy for ecological function, and the use of taxonomic identities to link to these established bodies of ecological knowledge. This approach is limited by the fact that many taxa detected through high-throughput sequencing lack experimentally verified ecological or functional traits, and by the ambiguity that results from reclassifications of taxonomic groups over time, including a recent revision of prokaryote nomenclature [7]. Resources are needed that move beyond dependence on individual studies and literature review, allowing ecological interpretations and assumptions about microbiome patterns to be tested directly. In this context, reverse ecology offers a promising approach that centers on the relationships between genetic sequences, their environmental distributions, and their *in situ* functional associations.

Reverse ecology is an approach that infers the ecological roles and traits of microbes from observed patterns in environmental sequence data, such as the distribution of genes, genomes, or taxa, across environmental gradients, host associations, or experimental conditions [8, 9]. Rather than focusing on characteristics of an organism, reverse ecology begins with a context, such as a soil disturbance or host, and identifies the genes, transcripts, operons, regulatory elements, or genomes that are consistently associated with it. This strategy leverages the power of DNA sequencing not just to detect presence, but to identify environment-wide associations and ecological signals in large volumes of microbiome data. Recent studies have used reverse ecology to highlight the roles of rare microbes in adaptation to diverse abiotic factors [10] and functional genes in relation to soil properties [11], and to associate specific bacterial taxa with responses to tillage and irrigation regimes in agricultural soils [12]. This approach is particularly well suited to pattern detection methods, such as machine learning [13], and gains further power when paired with experimental strategies like stable isotope probing (SIP), which directly link sequence data to in situ metabolic activity [9, 14]. SIP functions as a distinct, activity-based form of reverse ecology in which the inference of ecological role is grounded not in distributional patterns alone, but in direct experimental evidence of substrate assimilation, linking sequence identity to metabolic activity under environmentally relevant conditions. Overall, the effectiveness and scalability of reverse ecology is enhanced by the availability of curated, context-rich sequence databases [15–18], resources that may support training of predictive algorithms.

Nucleic acid SIP provides a powerful experimental complement to reverse ecology by directly linking specific microbial sequences with functional activities in natural contexts [19, 20]. In a SIP experiment, environmental samples (e.g. soils, sediments, or hosts) are supplied with an isotopically labeled “heavy” substrate (e.g. ^13^C, ^15^N, or ^18^O-enriched isotopologs). Organisms that metabolize the labeled substrates, or their by-products (i.e. “cross-feeding”), incorporate the heavy isotopes into their nucleic acids, which can then be separated based on their buoyant density shift and sequenced to designate “incorporators”: amplicon sequence variants (ASVs) or taxa [21–25]. This technique circumvents the need for inferring culture-based or taxonomic traits and instead provides a direct link to metabolic activity, though caveats do apply (see Current Limitations section). SIP experiments have revealed the ecological roles of previously uncultivated microbes in processes such as degradation of complex organic matter [26], microbial growth and turnover [27, 28], and trophic interactions like predation [22, 29]. The cross-study reuse has underscored the potential of aggregated SIP data to reveal broader ecological patterns [29]. Our research demonstrates how a curated database of nucleic acid SIP sequence data (“SIPdb”) can enhance the efficacy of a reverse ecology approach by linking microbial taxa to isotope incorporation patterns that reflect metabolic activity and ecological associations under environmentally relevant conditions.

Here, we describe the development and validation of the SIPdb, a comprehensive and extensible database designed to synthesize SIP-derived amplicon data for designating isotope incorporators across diverse substrate isotopolog experiments. The SIPdb accommodates three primary types of nucleic acid SIP gradient fractionation strategies: simple binary (“heavy” vs. “light”), position-resolved, and density-resolved; and two isotope incorporator designation strategies: fixed- and sliding-window. In curating the SIPdb, we adopted the Minimum Information for any Stable Isotope Probing Sequence (MISIP) standard [30], ensuring future SIP data can be easily integrated. While the current release is based on V4-region 16S rRNA gene data, the SIPdb framework can be extended to accommodate full-length amplicons and whole-metagenome SIP datasets, allowing for greater genomic resolution as more sequencing data become available. To accompany the SIPdb, we developed an interactive RShiny dashboard (www.sip-db.com) that enables researchers to explore, compare, and analyze their own microbiome data against curated SIP datasets. The dashboard supports users to perform sequence homology and taxonomy-based queries, along with data visualization. While SIPdb is not a quantitative SIP (qSIP) resource and does not provide absolute incorporation rates [31], it accommodates the most commonly generated nucleic acid SIP data types. Most importantly, it was designed to extend ecological interpretation to non-SIP microbiome datasets, offering a scalable and empirically grounded framework for inferring putative ecological associations and metabolic participation from SIP-derived sequence data, as demonstrated in our study.

## Materials and methods

### Obtaining and curating SIP amplicon sequencing datasets

The initial release of SIPdb integrates data from 22 SIP studies encompassing 21 amended isotopologs and 14 distinct environmental biomes (Table S1). Metadata from all included studies were standardized following the MISIP framework (Table S2), ensuring interoperability and facilitating cross-study integration. In total, the database comprises 4770 amplicon sequencing libraries and 163 417 unique ASVs, of which 46 484 were designated as significant isotope incorporators in one or more datasets (including H_2_^18^O) under the most relaxed criteria (log_2_FC > 1, *p*_adj_ ≤ 0.05). At present, only amplicon data overlapping the full V4 region of the 16S rRNA gene were kept, bounded by the primer set 515F (5′-GTGYCAGCMGCCGCGGTAA-3′) and 806R (5′-GGACTACHVGGGTWTCTAAT-3′) [32]. Raw sequence data, from each study, were processed independently in QIIME2 (v. 2024.10) [33] and taxonomic classification was assigned using the SILVA 138.2 database [34]. The ASV table, taxonomic assignments, representative sequences, sample metadata, and phylogenetic tree for each study were imported into R (v4.4.1) [35] and formatted into a phyloseq object (v1.48.0) [36]. Detailed information regarding data acquisition from the NCBI Short Read Archive, the specific parameters of the QIIME2 and DADA2 processing workflows, and the adaptive thresholds used for sparsity and quality filtering is available in the Supplementary Methods.

### Approaches to SIP incorporator designation

We developed a largely automated pipeline to standardize the identification of isotope incorporators from nucleic acid SIP datasets based on the differential abundance of sequences, either ASVs or taxa, in nucleic acid pools from isotopically enriched (“labeled”) and natural abundance (“unlabeled”) samples. The pipeline is coded in R and accepts phyloseq objects containing (i) an ASV count table, (ii) corresponding taxonomic assignments, and (iii) MISIP-formatted metadata including any other study factors. Importantly, the incorporator-designation pipeline does not use the phylogenetic tree or the representative ASV sequences; these elements are included in the phyloseq object only to support downstream analyses (e.g. UniFrac-based diversity metrics) and are not involved in determining incorporator status. Using SIP metadata and the count table, isotope incorporators are designated based on one of three gradient fraction cases and two window approaches for *in silico* combining of fractions. The pipeline outputs standardized results for each dataset, including: (i) taxon-level statistics for differential abundance (log_2_fold-change, *p*-values, adjusted *p*-values) generated by multiple methods (DESeq2 [37], edgeR [38], limma-voom [39], and ALDEx2 [40]), (ii) corresponding metadata describing incorporator designation strategy (heavy fraction, sliding window, or substrate-specific comparisons), and (iii) complete taxonomic and study-level provenance information. These results are compiled into a master table and summary reports that are subsequently imported into the SQLite SIPdb database (see next section). All code to reproduce the database is available on GitHub: https://github.com/alexbtrentin/sipdb-pipeline.

**Figure 1:**
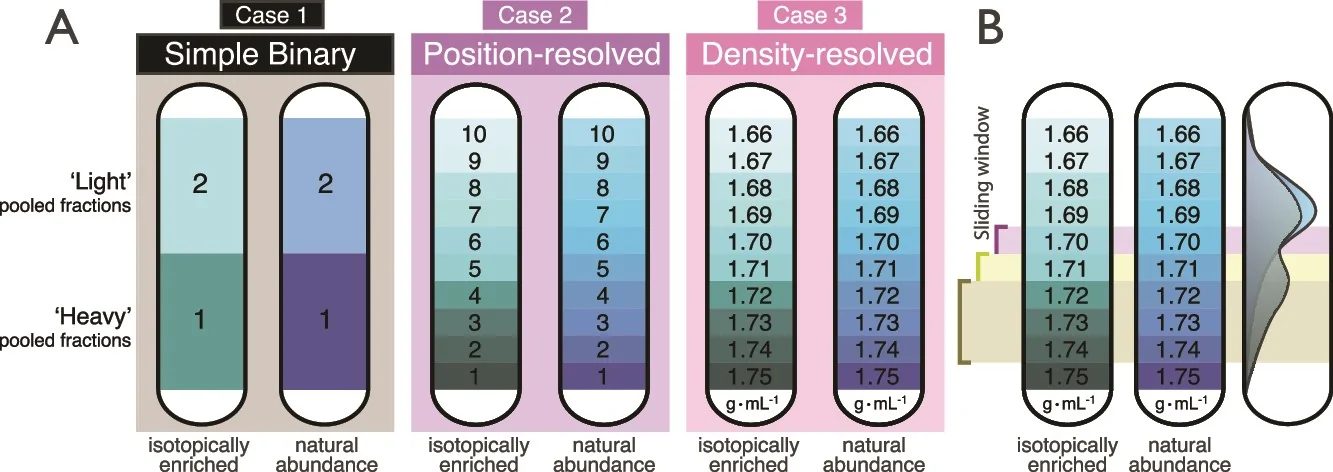
SIP gradient fractionation and windowing strategies used to designate isotope incorporators. In (A), the three SIP study types supported by SIPdb: Case 1 (simple binary comparison of pooled “light” and “heavy” fractions); Case 2 (position-resolved gradients with discrete fraction numbers); and Case 3 (density-resolved gradients with measured buoyant densities). In (B), the sliding-window approach applied to position- and density-resolved datasets, in which overlapping sets of adjacent gradient positions are compared between labeled and unlabeled samples.

To adapt to variation in SIP gradient fractionation approaches, the pipeline accommodates three different gradient structures when designating isotope incorporators: (case 1) “binary”, where fractions are pooled into heavy and light categories; (case 2) “position-resolved”, with multiple sequential fractions but no buoyant density information; and (case 3) “density-resolved”, where fractions include buoyant density data (Fig. 1A). To flexibly identify incorporators, the pipeline integrates four established differential abundance methods (DESeq2, edgeR, limma-voom, and ALDEx2) and applies two complementary strategies for contrasting differential abundance between labeled and unlabeled controls to designate incorporators: a fixed-window and sliding-window approach, as previously described [41].

Pooling fractions (*in silico*) along the SIP gradient can improve sensitivity for detecting isotope incorporators, particularly when individual fractions have low sequencing depth. Pooling is also essential for case 2 studies, where density information is lacking and corresponding positions may differ slightly in buoyant density. By aggregating adjacent gradient positions, contrasts between labeled and unlabeled samples become less affected by stochastic variation and yield more reliable statistical inference. We implemented two window-based pooling strategies: a fixed-window approach, which designates the heaviest fraction range based on a consistent proportion of available positions within each study, and a sliding-window approach, which sequentially compares sets of adjacent gradient positions across the full gradient (Fig. 1B). We validated and compared fixed-window, sliding-window, and adaptive k-nearest-neighbor approaches across diverse datasets to determine the most reliable strategy for identifying isotopically labeled taxa with full details on these validation procedures and statistical calculations provided in the Supplementary Methods.

### Validation of isotope incorporator designation with synthetic microbiome data

We evaluated the sensitivity, precision, specificity, and overall accuracy of incorporator designation using synthetic SIP datasets generated with SIPSim [42]. One hundred independent SIPSim simulations were generated using genome-derived GC-content distributions and defined isotope-incorporation status. Each simulation (*n* = 100 datasets) was analyzed using all three gradient fractionation strategies: binary, position-resolved, and density-resolved. Synthetic communities varied in richness, with 100, 150, or 219 taxa per simulation, and in the proportion of true incorporators, with 2%, 5%, 10%, 15%, 18%, or 30% of taxa designated as isotope incorporators. Isotope incorporation intensity was varied across simulations using atom-percent enrichment levels of 25%, 50%, 75%, or 100%. Each simulation included four isotope-labeled libraries and four natural-abundance control libraries. Their expected distribution across CsCl buoyant-density gradients was modeled with SIPSim, and the resulting fraction-level count tables were subjected to simulated PCR amplification and sequencing-depth subsampling. Ground truth was validated by confirming that all designated incorporators shifted in all labeled libraries and that no non-incorporator taxa exhibited isotope-induced buoyant-density shifts. Each synthetic dataset was processed through our complete analysis pipeline to assess the sensitivity, precision, specificity and F1 score (details in Supplementary Methods).

### Validation against original SIP studies

To further evaluate the accuracy of SIPdb, we compared the designated incorporator results from our pipeline against those reported in the original study describing each dataset (*n* = 22 studies). For all included studies, we systematically reviewed the corresponding manuscripts and supplementary materials to extract the primary isotope-incorporating taxa reported by the authors. When available, ASV-level sequence identifiers were directly retrieved. Otherwise, genus- or family-level incorporators were compiled from tables, figures, or text descriptions. These author-reported incorporators were assembled into a curated reference list spanning 411 taxa across 21 isotopologs, which served as the basis for validation against SIPdb results (Table S3).

We then queried SIPdb to determine whether each reported ASV/taxon was detected as a significant incorporator (*p*_adj_ ≤ 0.05, log_2_FC > 1) using any of the four differential abundance methods, given the variability of approaches used among studies. A taxon was considered “validated” if it appeared in the SIPdb results for the corresponding isotopolog and study, regardless of which method(s) detected it (Table S3). For validated taxa, we recorded which methods identified the incorporator and the mean log_2_FC value. When available, ASV-level identifiers or representative sequences reported in the original publications were directly used for validation against SIPdb results. When ASV-level information was not provided, validation was performed at the taxonomic rank reported by the authors (typically genus or family). A reported taxon was considered validated if it appeared as a significant incorporator in SIPdb for the corresponding study and isotopolog using any differential abundance method. This mixed-resolution validation reflects reporting practices in the original studies and avoids excluding datasets lacking ASV-level identifiers.

### Constructing the SIPdb relational database

For the initial release of SIPdb, we compiled the SIP results into a relational SQLite database that links differential abundance outcomes to study metadata, taxonomic lineages, and representative sequences. Incorporators were designated using both relaxed (≥1.70 g·mL^−1^) and stringent (≥1.725 g·mL^−1^) density thresholds to provide flexible sensitivity levels, with consensus calls derived from the integration of multiple statistical frameworks and windowing strategies. To ensure data integrity, we implemented a rigorous validation pipeline and integrated a curated BLAST database for sequence homology-based queries. Comprehensive details regarding the database architecture, filtering criteria, and validation protocols are provided in the Supplementary Methods.

### SIPdb analysis dashboard

**Figure 2:**
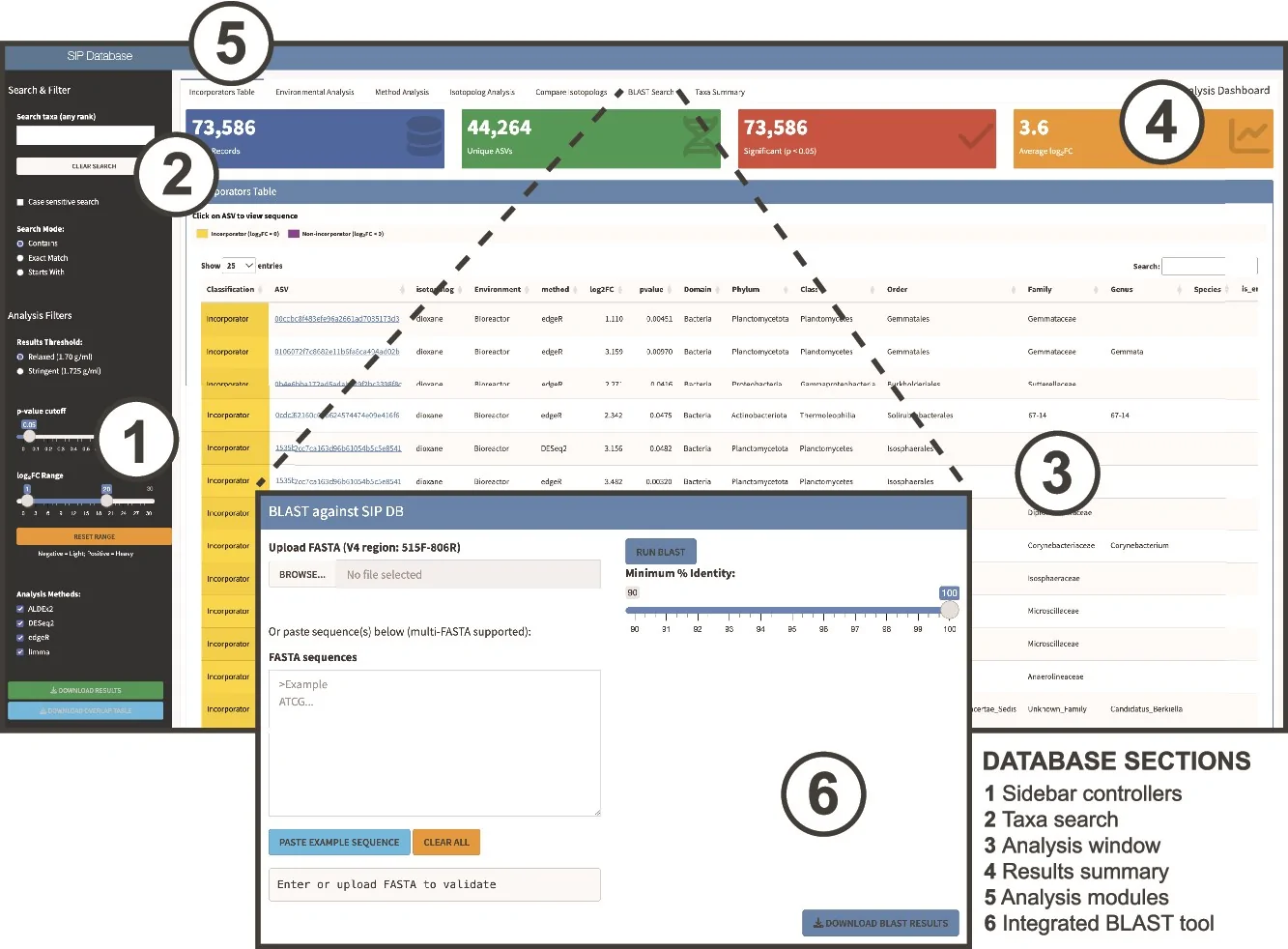
The SIPdb analysis dashboard. Screenshot of the interactive RShiny web application (www.sip-db.com) developed for querying and visualizing SIPdb data. Numbered components correspond to the labels printed on the figure: (1) sidebar controls for filtering results by density threshold (relaxed vs. stringent), *p*-value and log_2_FC cutoffs, and differential abundance method; (2) taxa search field for querying records by taxonomy at any rank, including ASV ID; (3) analysis window displaying the incorporators table, in which each ASV is shown with its differential abundance statistics (log_2_FC, *p*-value), taxonomic classification, isotopolog, environment, and method; (4) summary panels showing study and dataset metrics; (5) analysis modules navigation bar providing access to the dashboard’s seven modules; and (6) integrated BLAST tool for matching user-provided 16S rRNA gene V4 sequences (515F–806R) against SIPdb incorporator sequences. Together, these components enable interactive exploration of isotope incorporation data and direct annotation of users’ own microbiome datasets.

To facilitate interactive exploration and broad accessibility, we developed a comprehensive Shiny web [43] application that serves as the analysis dashboard for SIPdb (Fig. 2), which is available to users at www.sip-db.com. The dashboard allows users to explore isotope incorporators across diverse isotopolog substrates and environmental contexts, and to annotate their own microbiome datasets using a web-based BLAST search against representative sequences of incorporator ASVs in SIPdb. Users can adjust statistical thresholds (*p*-value cutoff, log_2_FC ranges, relaxed vs. stringent density thresholds) and compare differential abundance methods. The SIPdb analysis workflow (phyloseq objects and R scripts), the SQLite database and sequence data, and the dashboard app (“app.R”) are all open source and available for local use or adapting for future uses cases via GitHub (https://github.com/alexbtrentin/sipdb-dashboard). The dashboard comprises seven analysis modules: *Incorporators Table*, *Environmental Analysis*, *Method Analysis*, *Isotopolog Analysis*, *Compare Isotopologs*, *Taxa Summary*, and *BLAST Search*, which enable users to visualize community patterns, compare substrate-specific enrichment, and annotate sequences against the database. Comprehensive descriptions of module features are provided in the Supplementary Methods.

### Ecological inference with SIPdb

SIPdb can be used in two primary ways to derive ecological insights: (i) conducting meta-analyses across SIP datasets and (ii) mapping patterns of isotope incorporation onto non-SIP microbiome data to infer potential ecological associations or metabolic roles. To illustrate the first case, we visualized how substrate chemical properties influence microbial incorporation patterns across SIPdb by performing principal component analysis integrating substrate chemistry with incorporation metrics. For each substrate, we compiled six variables: (i) three chemical descriptors (solubility, hydrophobicity [xLogP3], and molecular weight from PubChem [44], Table S4) and (ii) three incorporation metrics from significant enrichment results (*p*_adj_ < 0.05): number of incorporating taxa (unique ASVs/OTUs), mean differential abundance in isotopically-enriched DNA or “effect size” (log_2_FC), and taxonomic breadth (number of unique phyla, a proxy for metabolic generality). Polymeric substrates (e.g. cellulose, DNA, chitin) were excluded because molecular descriptors are undefined for macromolecules with variable chain lengths, and substrates with incomplete chemical annotation were also excluded, yielding a total of 14 substrates in our analysis. Count-based variables were log_10_-transformed to address right-skewed distributions, and PCA was performed on scaled and centered data using prcomp in R [35]. To examine relationships between substrate chemistry and labeling strength, substrates were classified into effect-size categories (Low, Medium, High) based on tertiles of mean log_2_FC, and differences in PC1 scores among categories were assessed using Kruskal-Wallis tests with Dunn’s post-hoc comparisons (Benjamini-Hochberg correction).

To illustrate the second case, we evaluated whether SIPdb could aid interpretation of non-SIP microbiome datasets. We re-analysed 16S rRNA gene data from Li et al. (2025) [45], which examined two 1,4-dioxane-contaminated field sites with contrasting degradation outcomes (BioProject: PRJNA1183415). Data were processed using the same workflow applied to SIPdb studies. Briefly, raw reads were processed in QIIME2 (v. 2024.10) using DADA2 for denoising and chimera removal, yielding 13 176 ASVs, and taxonomy was assigned using SILVA 138.2 [34]. Putative 1,4-dioxane-responsive taxa were identified based on differential abundance (assessed using DESeq2, consistent with the analytical framework applied in the original study) between microcosms amended with 1,4-dioxane and unamended controls. Differentially abundant ASVs were queried against SIPdb using the integrated BLAST tool; however, few met the criterion of 100% identity across the full V4 region. In contrast, many ASVs that were not differentially abundant exhibited perfect sequence matches to SIPdb records. Consequently, a taxonomy-based matching approach was adopted. Differentially abundant ASVs from Li et al. were aggregated at the genus level, and genera were considered SIPdb-associated if any ASV classified to that genus had a matching record in SIPdb. Genera were then classified into three categories: *validated* (reported by Li et al. and present in the SIPdb match set), *recovered* (not reported by Li et al. but present in SIPdb as dioxane or phenanthrene incorporators), or *absent* (enriched in contaminated samples but not represented in SIPdb). SIPdb matches associated with other substrates (e.g. methane, butane, acetate) were retained for interpretive context only. Statistical comparisons used Wilcoxon tests for relative abundance and Fisher’s exact tests for presence-absence.

## Results and discussion

### Overview of SIPdb incorporator designations

Version 1 of the SIPdb currently contains 22 studies, representing a broad range of environments (14 different environment types) and substrates (21 isotope labelled substrates). The database identified 46 484 unique ASVs as significant isotope incorporators across all individual differential abundance methods (*p*_adj_ ≤ 0.05, log_2_FC > 1), of which 33 939 were retained as consensus incorporators (≥ 2 methods). The isotopolog substrates span a broad range of metabolic contexts, including simple carbon substrates (glucose, methane), fermentation products (acetate, lactate, oxalate), complex carbohydrates (alginate, cellulose), aromatic compounds (phenanthrene, vanillin), and specialized substrates such as alkanes (butane), xenobiotics (dioxane, sulfamethoxazole), and biomolecules (DNA, amino acids). Within this set, ^18^O-labeled water occupies a distinct functional category, as it reflects the prevailing metabolic activity of all physiologically active organisms, independent of any specific substrate amendment. This allows ^18^O-H₂O to serve as a baseline indicator of the actively growing microbiome within each environmental context, while underscoring the need to interpret SIPdb-derived incorporation patterns in light of the distinct ecological meanings associated with different isotopologs.

**Figure 3:**
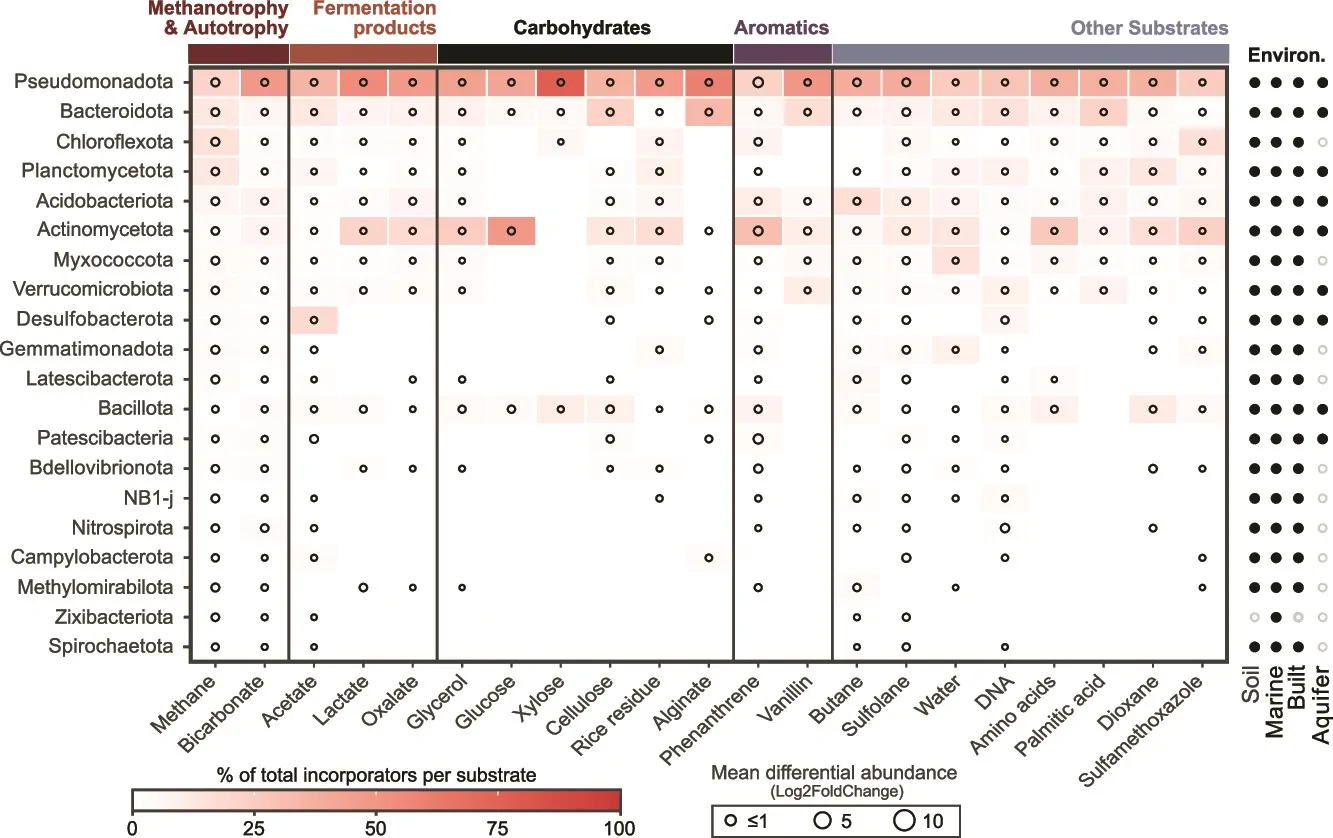
Distribution of isotope incorporators across substrates, phyla, and environments. Bubble heatmap summarizing isotope-incorporating ASVs for the 20 most prevalent phyla (rows) across the 21 labeled substrates (columns) in SIPdb. For each phylum–substrate combination, the square color shows the percentage of that substrate’s total designated incorporators assigned to the phylum and circle size corresponds to the mean differential abundance (log_2_FC). The right panel indicates the environment types in which each phylum was detected as an incorporator (soil, marine, built, aquifer). Filled circles denote detection and open circles non-detection.

The SIPdb incorporator set spanned 62 phyla, with members of Pseudomonadota (27.6%), Bacteroidota (11.0%), and Chloroflexota (9.8%) representing the most frequently detected incorporators across studies. The predominance of Pseudomonadota as incorporators highlights the broad metabolic versatility and successful ecological strategies of this diverse lineage of bacteria, with members designated as incorporators across diverse substrates (Fig. 3). Bacteroidota and Chloroflexota were also major isotope incorporators, though with a higher degree of substrate-specific associations.

Single-carbon (C1) substrates yielded the highest numbers of designated incorporators, reflecting both the ubiquity of C1 metabolism and the foundational role of these organisms in supporting carbon assimilation at higher trophic levels. Bicarbonate was incorporated by 3633 ASVs across 41 phyla (mean log_2_FC = 2.35), indicating that the capacity to assimilate inorganic carbon is phylogenetically widespread, encompassing ~7% of ASVs detected in SIPdb. This proportion is higher than, but broadly comparable to, genome-based estimates suggesting that ~2% of bacterial and archaeal genomes encode complete carbon fixation pathways [46]. Importantly, our result is likely an overestimate given it likely reflects a combination of primary autotrophy, mixotrophy, and downstream incorporation via cross-feeding and trophic transfer, processes that cannot be disentangled using SIP alone. Consistent with this interpretation, Chloroflexota exhibited strong methane incorporation (3624 ASVs), highlighting their potential importance within methanotrophic and methane-supported microbial communities [47]. Many methane studies in SIPdb spanned long incubation periods (24 to 720 h), which likely promote secondary consumption and food-web propagation of ^13^C, explaining the large apparent taxonomic breadth of methane incorporators despite the taxonomically narrow set of known primary methanotrophs. In contrast to C1 compounds, a relatively narrow set of phyla were designated as glucose incorporators in SIPdb, which is unexpected given that glucose uptake is broadly distributed among bacteria [48]. These observations suggest that growth traits (uptake kinetics and specific growth rates), substrate concentration, incubation time, and bioavailability, not just the presence of metabolic pathways, determine whether a taxon is designated as an incorporator [44, 49].

Together, the contrasting breadth of incorporators observed for C1 substrates versus glucose highlights the ecological and methodological complexity inherent to SIP-derived incorporator data, emphasizing that incorporator breadth in SIP experiments reflects ecological context and trophic dynamics rather than simple substrate-pathway relationships.

### Benchmarking incorporator designation with synthetic SIP datasets

**Figure 4:**
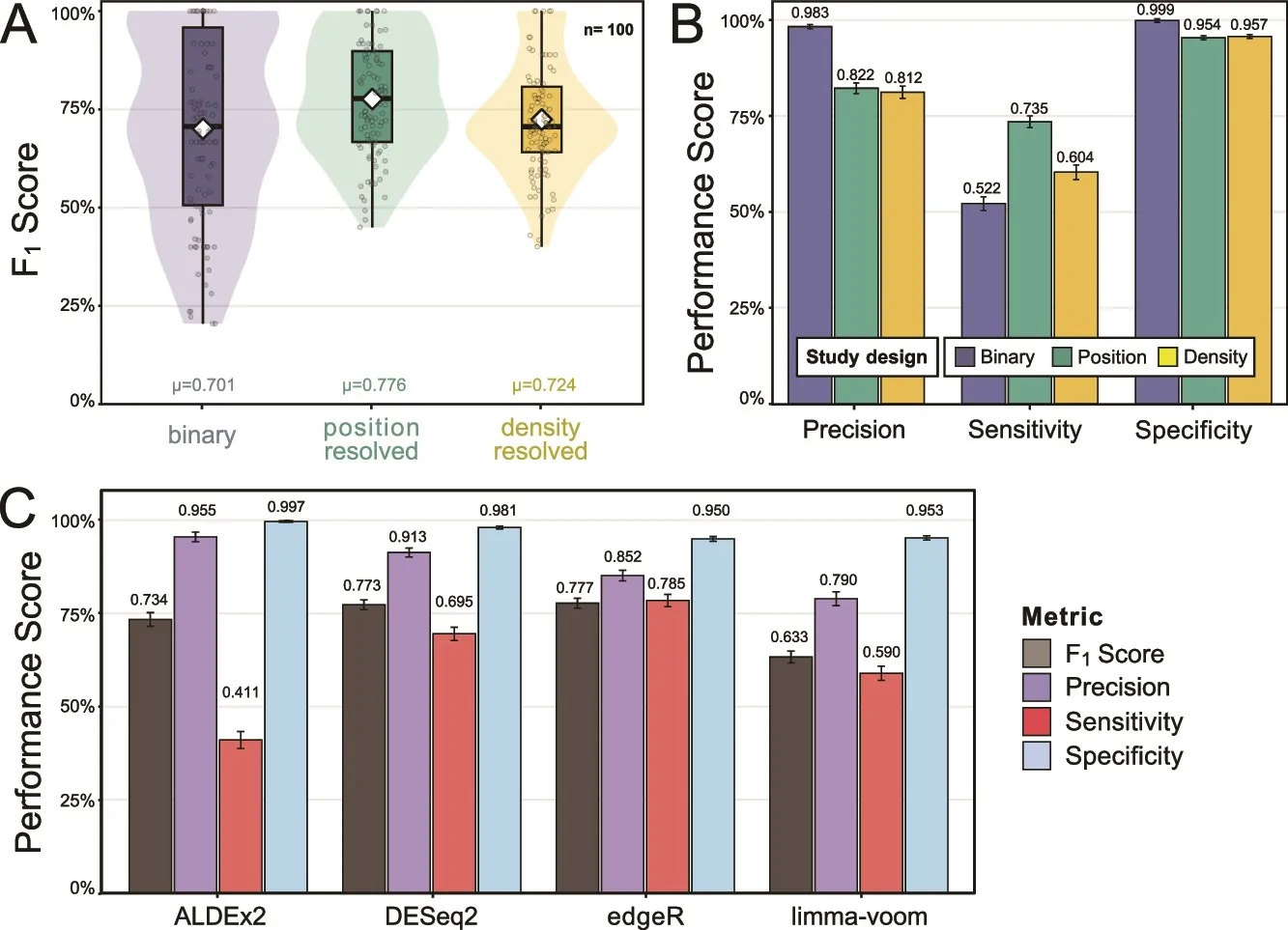
Benchmarking of gradient fractionation strategies and differential abundance methods using synthetic SIP datasets generated with SIPSim. In (A), violin plots with overlaid box plots showing the distribution of F1 scores for incorporator designation under each gradient fractionation strategy (binary, position-resolved, density-resolved; n = 100 synthetic datasets each). Boxes show the median and interquartile range, whiskers extend to 1.5× the interquartile range, and white diamonds mark the mean. In (B), mean precision, sensitivity, and specificity for each gradient fractionation strategy, colored by strategy. Values are printed above each bar. In (C), mean F1 score, precision, sensitivity, and specificity for each differential abundance method (ALDEx2, DESeq2, edgeR, limma-voom). Error bars denote standard error. Across all panels, performance is evaluated against the known incorporator status of taxa in the synthetic communities using the fixed-window strategy.

Across 100 synthetic SIP datasets generated with SIPSim, performance was shaped by both gradient fractionation strategy and differential abundance method. The position-resolved approach yielded the strongest mean F1 score (0.776), density-resolved was intermediate (0.724), and binary was the least effective in incorporator designation (0.701) (Fig. 4A and B). The binary strategy gave the most stringent results, with high precision (0.983) and specificity (0.999), recovering fewer than half of true incorporators (low sensitivity) while almost never producing a false call. The position-resolved strategy achieved the best balance of precision (0.822) and sensitivity (0.735), while the density-resolved strategy was intermediate (precision = 0.812; sensitivity = 0.604). The stronger performance of gradient-resolved strategies over binary pooling is consistent with prior benchmarking [42, 50]. Binary pooling carries the additional risk that natural variation in genome G + C content can shift the buoyant density of unlabeled DNA by up to ∼0.05 g·mL^−1^, allowing unlabeled to be miscalled as incorporated when position or density information is unavailable [41]. Resolved designs, which retain gradient structure, are less susceptible to this confounding.

Method choice and gradient design contributed comparably to variation (Fig. 4C, Table S5). Among the SIPSim datasets for which all methods returned results, edgeR and DESeq2 performed best and had comparable scores, with edgeR (F1 = 0.777, precision 0.852, sensitivity 0.785) favoring sensitivity and DESeq2 precision (F1 = 0.773, precision 0.913, sensitivity 0.695). ALDEx2 was the most precise (0.955) but least sensitive (0.411), giving an intermediate F1 (0.734), and limma-voom was weakest (F1 = 0.633). The methods also differed markedly in applicability, with DESeq2, edgeR, and limma-voom returning results for every dataset, whereas ALDEx2 generated results for only 67.7% overall (48% of binary, 67% density, and 75% position-resolved designs), reflecting a known limitation of its interquartile log-ratio normalization on sparse contrasts [40] (details in Supplementary Methods). We tested whether substituting the zero denominator on these contrasts could recover the missing calls: across the benchmark it recovered 360 additional true incorporators but added 406 false positives, with the cost concentrated in density-resolved designs, where precision fell from 0.74 to 0.47, while binary and position-resolved designs were little affected. We therefore retained the IQLR denominator for SIPdb version 1 results. No method or design was best across all performance metrics: resolved designs were most sensitive, edgeR and DESeq2 most accurate and broadly applicable, and ALDEx2 most precise but least widely usable.

With the current criteria (p_adj_ < 0.05, log2FC > 1), edgeR accounts for a majority of per-method incorporator calls for SIPdb study datasets. Two factors drive this: (i) edgeR was among the most sensitive methods in our benchmark (0.785) and (ii) other methods had limitations that reduced outputs, specifically, where ALDEx2 was able to compute only 43 of 113 contrasts (38%) using the default IQLR denominator approach. The overrepresentation of edgeR results reflects differences in statistical stringency and applicability, meaning the aggregated results of all four methods accessible via the SIPdb are valid, but should be interpreted as a discovery set rather than a confirmed inventory. For new SIP data, DESeq2 is a reasonable default for balanced designation, edgeR for broad discovery, and ALDEx2 for stringent calls on feature-rich contrasts. For filtering within the current release, stringency matters more than method choice: the union returns 46 484 ASVs (edgeR-dominated), the consensus subset (≥ 2 methods) 34 939 ASVs (recommended for hypothesis generation), and the strictest filter (≥ 3 methods) 3006 ASVs, yielding the highest likelihood of isotope incorporation. These results align with reports that method choice is a major source of variance in microbiome analyses [51].

### Benchmarking against the original SIP studies

To validate SIPdb results against published findings, we systematically extracted the primary incorporators reported by authors in each of the 22 original studies, resulting in 411 taxa spanning genus to phylum level across 21 distinct isotopologs (Table S3). Of these author-identified incorporators, 70.1% (288/411 rank-agnostic-taxa) were successfully detected in SIPdb using our standardized analysis pipeline, demonstrating strong concordance between the database and original study conclusions. Detection rates varied substantially by substrate: perfect validation (100%) was achieved for alginate (8/8 reported incorporators recovered) and DNA (6/6), with high rates for water (88.9%), glucose (88.9%), sulfamethoxazole (88.9%), bicarbonate (85%), butane (83.3%), and methane (81.1%).

Lower detection rates were observed for palmitic acid (7.7%, 1/13) and xylose (8.3%, 1/12 reported incorporators), both of which were reported in a single study in the current database. To determine whether these results reflected differences in statistical stringency rather than analytical failure, we performed a sensitivity analysis by relaxing the log_2_FC threshold to >0 while maintaining other criteria. Detection rates increased to 69.2% (9/13) for palmitic acid and 33.3% (4/12) for xylose, demonstrating that many author-reported incorporators produce detectable enrichment signals in SIPdb but with effect sizes below our conservative threshold.

Non-detection of author-reported incorporators (29.9%) likely stems from (i) statistical threshold differences between original studies and SIPdb’s standardized *p*_adj_ ≤ 0.05 criterion, (ii) taxonomic assignment variation across reference databases and algorithms, (iii) data processing heterogeneity in normalization and quality filtering, and (iv) biological factors including stochastic sampling of low-abundance taxa. The strong overall concordance (70.1%) demonstrates that SIPdb successfully reproduces most published SIP findings while maintaining conservative statistical rigor, supporting its utility as a reliable resource for comparative SIP analysis.

### Cross-study exploration of substrate-associated incorporator profiles

**Figure 5:**
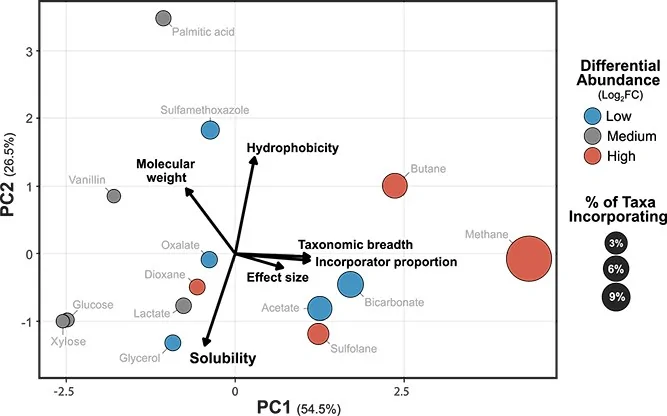
Principal component analysis relating substrate properties and incorporator patterns (n = 14 substrates). Biplot of a PCA in which each substrate is described by six variables: three physicochemical descriptors (solubility, hydrophobicity [xLogP3], molecular weight) and three incorporation metrics (effect size [mean log₂FC], taxonomic breadth [number of unique phyla], and incorporator proportion [proportion of detected ASVs designated as incorporators]). Each point represents a labeled substrate, colored by mean effect size (log2FC) and scaled by the proportion of ASVs designated as incorporators for that substrate relative to the total number of ASVs detected in the corresponding study or studies. Arrows show the loadings of all six variables. Polymeric substrates (e.g., cellulose, DNA, chitin) and those with incomplete chemical annotation were excluded.

To validate the ecological coherence of SIPdb and explore how substrate chemistry shapes incorporator communities, we performed a principal component analysis integrating substrate physicochemical properties with incorporation metrics across 14 substrates. As a first validation, we asked whether SIPdb could recapitulate the well-established expectation that small, soluble substrates support broader or more diverse microbial incorporator communities compared with large, hydrophobic, or recalcitrant substrates, which require specialized degradative machinery [52]. The second principal component (PC2, 26.5%) confirmed this expectation, with loadings associated with physicochemical accessibility separating small, highly soluble substrates (e.g. acetate, glycerol) from hydrophobic or high-molecular-weight compounds (e.g. palmitic acid, sulfamethoxazole). That SIPdb recovers this pattern from aggregated cross-study data provides confidence that the database faithfully captures substrate-driven ecological patterns (Fig. 5).

The more informative axis, however, was the first principal component (PC1, 54.5%), which exhibited strong loadings for the proportion of incorporator taxa (0.50), the total number of incorporators (0.50), and taxonomic breadth (0.48). Together, these metrics capture variation in trophic accessibility, reflecting the extent to which a substrate’s isotopic label propagates through the microbial food web, from primary consumers to downstream cross-feeders [53, 54]. Substrate positions along PC1 corresponded to significant differences in labeling strength (approximated by log_2_FC in relative abundance), where high-effect size substrates clustered at the high-trophic-accessibility end of PC1 (mean PC1 = 1.84), whereas medium-effect substrates occupied the low-trophic-accessibility region (mean PC1 = −1.73; Kruskal-Wallis χ^2^ = 8.73, *P* = 0.013). Notably, methane departs from this chemical expectation: despite being a small, non-polar gas, it is among the most broadly incorporated substrates in the database, reflecting the wide phylogenetic distribution of methane monooxygenase systems [55] rather than substrate lability. Together, these axes demonstrate that substrate identity structures microbial communities not only through chemical constraints on assimilation, but also through the extent to which isotopic label is accessed across trophic levels after primary assimilation, a pattern consistent with documented cross-feeding dynamics in SIP experiments [56–59].

To evaluate reproducibility across independent studies, we examined substrates tested multiple times. Mantel tests revealed a significant positive correlation between study replication and phylum-level similarity (Spearman *r* = 0.256, *P* = 0.026), indicating that substrates examined across diverse environments, including methane and cellulose, yielded more consistent incorporator communities than those from single studies. This pattern was not driven by differences in within-substrate variance (betadisper *P* = 0.729). Reproducibility was strongly scale-dependent, with phylum-level profiles exhibiting substantially higher overlap across studies (mean Jaccard similarity = 0.44 ± 0.21) than genus-level profiles (0.039 ± 0.05), an 11.3-fold difference. This scale dependence mirrors broader patterns in microbial ecology, in which high-level functional traits are conserved within major clades, while fine-scale taxonomic composition reflects local environmental filtering [60–62]. Practically, this suggests that SIPdb information is propagated across broad taxonomic ranks, with phylum- and class-level guilds providing more reliable guidance for identifying likely incorporators of novel substrates than genus-level assignments. Together, these results show that SIPdb captures generalizable ecological rules of substrate use that extend beyond primary metabolism to include trophic accessibility.

### Reverse ecology approach using SIPdb

To evaluate whether SIPdb can inform mechanistic interpretation of non-SIP microbiome datasets, we mapped 16S rRNA gene profiles from a study of 1,4-dioxane degradation (Li et al., 2025) [45] onto SIPdb and assessed whether enriched taxa had prior isotope-based evidence of substrate assimilation. SIPdb currently contains one dioxane-degradation SIP dataset, conducted in activated sludge bioreactors, whereas Li et al. examined degradation in polluted groundwater sediments. Our goal was to assess whether SIPdb could help identify patterns in isotope-incorporating taxa, revealing genera with documented incorporation capacity that might otherwise be overlooked in site-specific analyses. Importantly, any matches we observe are likely to reflect both primary dioxane-degrading populations as well as cross-feeding taxa whose incorporation is driven by trophic propagation.

**Figure 6:**
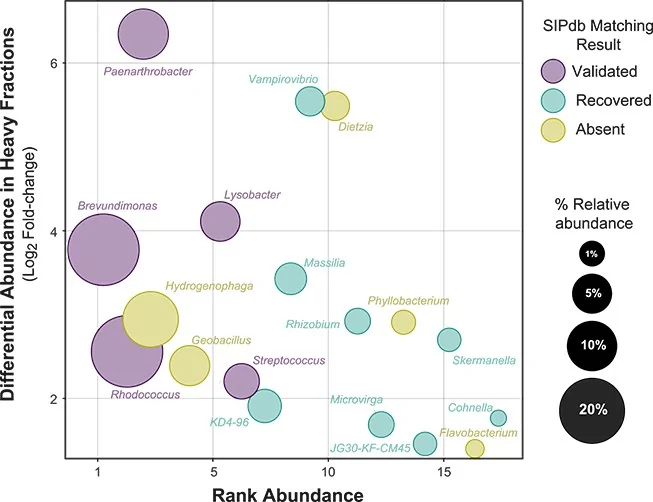
Reverse-ecology annotation of 1,4-dioxane-responsive taxa using SIPdb. Genera enriched in the 1,4-dioxane-amended microcosms of Li et al. (2025), a non-SIP dataset, plotted by abundance rank (x-axis; rank 1 = highest relative abundance) and differential abundance (y-axis; log2FC, dioxane-amended vs. unamended controls). Dot size indicates each genus’s mean relative abundance in the contaminated samples, and color indicates its SIPdb matching result: “validated” (reported by Li et al. as dioxane-responsive and matching a SIPdb record), “recovered” (enriched but not reported by Li et al., yet matching a SIPdb record), and “absent” (enriched but lacking a matching SIPdb record). Because exact OTU-level correspondence between datasets was rare, matching was performed at the genus level.

Exact OTU-level correspondence between datasets was rare, so a taxonomy-based approach was used to designate SIP incorporators. Of the 10 genera reported by Li et al. as enriched at dioxane-contaminated sites, five matched SIPdb records indicating incorporation by dioxane or the structurally related compound phenanthrene (Fig. 6). These validated taxa were not randomly distributed within the community; they included several of the most abundant populations, such as *Brevundimonas* (26.1% of total reads, log_2_FC = 4.1), *Rhodococcus* (25.1%, log_2_FC = 2.7), and *Paenarthrobacter* (log_2_FC = 7.2; 9.8%). This pattern suggests that even with limited dioxane-specific data, SIPdb can help identify ecologically dominant degraders relevant to bioremediation applications. The validation of *Brevundimonas* and *Streptococcus* through direct dioxane SIPdb matches, alongside *Rhodococcus, Paenarthrobacter,* and *Lysobacter* through phenanthrene records, demonstrates the value of warehousing structurally related compounds in the SIPdb as means for discovery. The inclusion of phenanthrene-incorporating taxa in our query reflects the observation that bacteria capable of PAH degradation often possess diverse catabolic capabilities and broad-substrate monooxygenase systems [63–65].

Five differentially abundant genera in Li et al. dioxane-amended microcosms were absent from SIPdb’s dioxane/phenanthrene records: *Dietzia, Hydrogenophaga, Phyllobacterium, Geobacillus,* and *Flavobacterium*. These taxa played an important role in the study system, with *Dietzia* exhibiting the second-highest relative abundance increase in their experiment (log_2_FC = 5.7), while *Hydrogenophaga* was among the most abundant (13.6% of total reads). Notably, three of the absent genera, *Hydrogenophaga, Flavobacterium*, and *Geobacillus*, were identified as incorporators for methane or butane incorporation in the SIPdb. This observation has important mechanistic implications: soluble di-iron monooxygenases, including propane monooxygenases and butane monooxygenases, exhibit broad substrate specificity and are well-documented for the cometabolic oxidation of dioxane [66, 67]. Taxa capable of C2-C4 alkane oxidation may therefore possess latent dioxane degradation capacity through these promiscuous enzyme systems [68]. This result demonstrates how SIPdb may extend interpretation of results, enabling hypothesis generation beyond simple substrate matching. Querying taxa enriched for related pathways (e.g. C2-C4 alkane oxidation) allows users to infer co-metabolic potential even when direct SIP evidence is unavailable. Regardless, users may anticipate that phylotypes and taxa key to their study system may be absent from SIPdb due to either gaps in database coverage or geographical and ecological differences.

Beyond confirming known degraders, SIPdb matches identified eight genera with documented dioxane and phenanthrene incorporation that were enriched at other contaminated sites but not reported in Li et al. Recovered genera included *Vampirovibrio* (log_2_FC = 5.8), Massilia (log_2_FC = 3.5), and multiple *Rhizobiales* members, including *Rhizobium, Skermanella*, and *Microvirga*. The presence of multiple Rhizobiales is notable given this order’s known metabolic versatility [69, 70]. *Vampirovibrio*, a predatory bacterium, may represent indirect enrichment through predation on primary degraders rather than direct dioxane metabolism. This fact illustrates the importance of interpreting matches to the SIPdb with caution, as the SIPdb can reveal both direct incorporators and taxa responding indirectly to contaminant exposure [29, 71]. Our reverse ecology approach demonstrates the use of SIPdb to drive hypothesis generation.

### Future advances and the promise of the SIPdb

Despite the relatively limited number of publicly available datasets in the SIPdb, due to legacy issues in data curation [30], our experimentation with the initial release of SIPdb revealed patterns in microbial substrate incorporation and demonstrated its promise as a tool for reverse ecology. All SIPdb entries are formatted according to the MISIP standard [30], ensuring its forward compatibility with newly generated SIP datasets. MISIP is slated for adoption as a formal MIxS checklist, which will guide SIP data archival for integration in the SIPdb. Furthermore, the volume of SIP sequencing data is expected to grow as recent innovations in high-throughput, semi-automated workflows reduce the labor burden associated with SIP, cutting hands-on time by six-fold [56]. Previous advances in SIP computational methods were integral to the development of the SIPdb (e.g. HTS-SIP [50]) and continued progress in this space, including the use of synthetic DNA internal standards for improved comparability and accuracy [72], will enhance SIPdb’s utility. As a fully open-source project, SIPdb is designed to support and grow with this evolving ecosystem, serving as a scalable platform for meta-analysis, cross-study synthesis, and hypothesis generation in microbial ecology.

### Current limitations and methodological evolution

While SIPdb represents a step forward in enabling cross-study synthesis and application of reverse ecology with SIP-derived sequence data, several limitations of its current form should be acknowledged. First, the database is currently restricted to V4 region amplicon data which has limited phylogenetic resolving power. This limits its utility for identifying fine-scale functional variation, such as strain-level population dynamics or accessory gene content, which are increasingly recognized as important drivers of microbial ecology and biogeochemical function [73–75]. Expanding the SIPdb to include shotgun metagenomic and metatranscriptomic datasets will be essential for improving both taxonomic resolution and direct functional inference. However, such data were still relatively scarce at the time of publication. The SQLite database and RShiny dashboard were designed with modularity and extensibility in mind, allowing users to apply different differential abundance frameworks and customize enrichment detection strategies.

One of these frameworks is quantitative SIP (qSIP), which estimates atom-percent excess from per-fraction gene copy numbers [31]. Although the HTS-SIP framework used in our pipeline supports qSIP [50], incorporator designation is sufficient for the cross-study reverse-ecology applications SIPdb targets. Most users need to know which taxa incorporated a given substrate, not the relative rates at which they did so. Quantitative atom-percent excess incorporation rates are most informative for within-experiment comparisons among co-occurring populations, a distinct analytical goal better addressed by dedicated qSIP tools. Consequently, the SIPdb is well appointed to serve the majority of the SIP community, where conventional SIP accounts for roughly 94% of the SIP literature (qSIP ~6%; Fig. S1). The current BLAST-based search architecture may need to evolve toward more scalable alignment and indexing tools, as well as more platforms with greater computational resources like Galaxy [76] or KBase [77].

An additional limitation lies in the abstraction of study-specific methodological detail into simplified categorical metadata fields used in designating the isotopic incorporators that form the basis of the SIPdb. While necessary for interoperability, this standardization introduces a degree of data loss, particularly around incubation conditions, substrate labeling strategies, and gradient fractionation schemes. These factors can influence the identity and detectability of incorporators, potentially biasing results presented in the SIPdb toward taxa exhibiting the strongest incorporation effects. This tradeoff between standardization and preserving useful contextual information reflects a broader challenge in microbiome database development, where ease of use and broad applicability must be balanced against preserving nuanced experimental context. Future iterations of SIPdb could include layered metadata or filterable provenance flags that allow expert users to drill down into methodological differences while retaining an accessible default view for simple exploratory analyses.

## Supplementary Material

Supplementary_material_ycag216

## Data Availability

The SIPdb analysis workflow (phyloseq objects and R scripts), the SQLite database and sequence data, and the dashboard app (“app.R”) are all open source and available for local use or adaptation to future use cases via GitHub (https://github.com/alexbtrentin/sipdb-pipeline). All additional study metadata are summarized in Table S2. SIPSim simulation information is summarized in Table S6 (reference genome inputs), Table S7 (simulation design parameters), and Table S8 (run settings). Intermediary files used for SIPdb analysis and queries are archived on The Open Science Framework (OSF) under the accession: https://osf.io/cwg6r/overview.
