# Supplementary material for: SIPdb: a stable isotope probing database and analytical dashboard for linking amplicon sequences to microbial activity using reverse ecology
Source: ISME Commun. 2026 Jul 28;6(1):ycag216. doi: 10.1093/ismeco/ycag216 (PMC13518055; doi:10.1093/ismeco/ycag216)

Figure S1. Conventional stable isotope probing methods remains the predominant SIP approach compared to quantitative SIP (qSIP). Annual publication counts (2000–2026) for stable isotope probing (SIP; blue bars) and the subset using qSIP (orange dashed line) compiled from Web of Science and Scopus (1,131 records total: 1,069 SIP and 62 qSIP), indicating that qSIP accounts for roughly 6% of the SIP literature. SIP records were identified using the topic query ("stable isotope probing" OR "DNA-SIP" OR "DNA stable isotope probing") and qSIP records ("quantitative stable isotope probing" OR "qSIP" OR "quantitative SIP" OR "DNA-qSIP"). The dashed vertical line marks the introduction of qSIP (Hungate et al., 2015). Hatched bar and open diamond marker indicate partial counts for 2026.

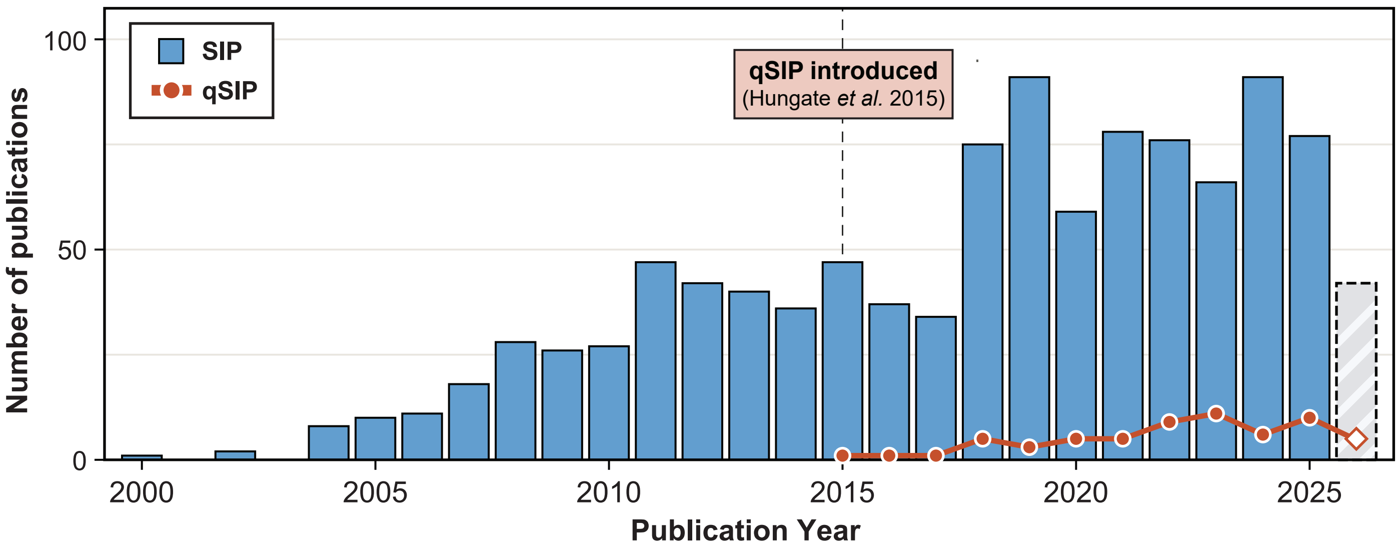

Supplement: Supplementary file 1 [file supplementary_material_ycag216.zip › Trentin et al. (2026) - Supplementary Figures - final.pdf]
