# Supplementary material for: SIPdb: a stable isotope probing database and analytical dashboard for linking amplicon sequences to microbial activity using reverse ecology
Source: ISME Commun. 2026 Jul 28;6(1):ycag216. doi: 10.1093/ismeco/ycag216 (PMC13518055; doi:10.1093/ismeco/ycag216)
Supplement: Supplementary file 1 [file supplementary_material_ycag216.zip › Trentin et al. (2026) - Supplementary Methods - final.docx]

**SUPPLEMENTARY METHODS**

SIPdb: A stable isotope probing database and analytical dashboard for linking amplicon sequences to microbial activity using reverse ecology

Trentin *et al.*, 2026

*Sequence processing and phyloseq object construction*

The initial release of SIPdb integrates data from 22 SIP studies encompassing 21 amended isotopologs and 14 distinct environmental biomes (**Table S1**). In total, the database comprises 4,770 amplicon sequencing libraries and 163,417 unique ASVs, of which 46,484 were designated as significant isotope incorporators in one or more datasets (including H_2_^18^O) under the most relaxed criteria (log_2_FC > 1, *p*_adj_ ≤ 0.05). Incorporator criteria can be adjusted to implement more conservative threshold to filter SIPdb associations (as described below). Candidate datasets were identified through a combination of searches in the NCBI Short Read Archive (SRA), peer-reviewed literature, and a curated compilation provided by Simpson et al. (2023). Raw sequencing files were retrieved from the SRA using fasterq-dump from the SRA Toolkit [1] and processed through a standardized QIIME2 workflow to generate phyloseq objects for downstream analysis (details in Supplementary Methods).

Due to incomplete study metadata (see ref [2]), a total of 22 high-quality SIP datasets met all inclusion criteria and were retained in SIPdb. For inclusion in the SIPdb (current and future), all datasets must have the minimum set of metadata required by MISIP: isotope, isotopolog, isotopolog label, isotopolog approach, and gradient position. To ensure the initial instantiation of the SIP was exemplary, we added quality thresholds stipulating that all datasets contain: more than one gradient position and comply with sequencing depth and sparsity quality thresholds (see next paragraph). Metadata from all included studies were standardized following the MISIP framework (available in **Table S2**), ensuring interoperability and facilitating cross-study integration.

At present, only amplicon data overlapping the full V4 region of the 16S rRNA gene was kept, bounded by the primer set 515F (5'-GTGYCAGCMGCCGCGGTAA-3') and 806R (5'-GGACTACHVGGGTWTCTAAT-3'). [3] Raw sequence data, from each study, were processed independently in QIIME2 (v. 2024.10), [4] with quality filtering, denoising, and chimera removal performed using the DADA2 plugin (q2-dada2 version 2024.10.0), producing ASVs. DADA2 error models were learned separately for each study. Taxonomic classification was assigned against the SILVA 138.2 database. [5] The resulting feature table was filtered to remove sparse and low-abundance taxa. ASVs were retained if they: (i) had at least 3 total counts across all original study samples, (ii) were present in at least two study samples or 1% of samples per study, and (iii) exhibited ≤99% sparsity across study samples. Additionally, samples with fewer than 1,000 total reads were excluded from differential abundance testing, along with the paired controls. These adaptive thresholds balance retention of rare taxa that may become isotope incorporators with removal of potential sequencing artifacts, while accounting for varying sample sizes across the 22 studies. Sequences identified as chloroplasts or mitochondria were removed. A rooted phylogenetic tree was generated to support downstream diversity analyses using the QIIME2 q2-phylogeny workflow align-to-tree-mafft-fasttree. Finally, the ASV table, taxonomic assignments, representative sequences, sample metadata, and phylogenetic tree for each study were imported into R (v4.4.1) [6] and formatted into a phyloseq object (v1.48.0). [7]

*Gradient handling and window definition*

Studies were programmatically classified into one of three gradient structures based on available metadata: (case 1) binary gradients with pooled heavy and light fractions; (case 2) position-resolved gradients lacking buoyant density information; and (case 3) density-resolved gradients with measured buoyant densities. For position-resolved studies (case 2), a fixed-window approach grouped the heaviest 33% of gradient positions from labeled samples and contrasted them with the corresponding positions from unlabeled controls, excluding lighter fractions from both gradients, following Youngblut et al. (2014) [8]. Sliding-window analyses sequentially compared adjacent positions across the gradient; studies with ≤3 fractions were analyzed at the single-fraction level, whereas studies with ≥5 fractions used hierarchical clustering (Ward’s method with Euclidean distance) [9] to define two to five windows based on positional proximity.

For density-resolved studies (case 3), fixed windows were defined using buoyant density thresholds of ≥1.725 g mL⁻¹ for stringent analyses and ≥1.700 g mL⁻¹ for relaxed analyses, with optional intermediate windows (1.710–1.725 g mL⁻¹). Sliding-window analyses for density-resolved gradients used continuous density values directly and did not require clustering. An optional k-nearest-neighbor (KNN) strategy [13] selected equal numbers of labeled and unlabeled samples (default: k = 3 per group, adaptively reduced when sample numbers were limited) nearest to each gradient position.

*Statistical integration and incorporator criteria*

Within each window, differential abundance between isotopically labeled and unlabeled samples was tested independently with four methods, each applied with a labeled-versus-unlabeled design and Benjamini-Hochberg correction. DESeq2 used median-of-ratios normalization and the Wald test under a parametric dispersion fit, with a local fit applied as a fallback where the parametric model failed to converge. edgeR used TMM normalization, robust tagwise dispersion estimation, and the quasi-likelihood F-test, retaining features with counts-per-million above 0.5 in at least two samples. limma-voom used TMM normalization with observation- and sample-level precision weights (voomWithQualityWeights) and empirical Bayes moderation, with features filtered by filterByExpr. ALDEx2 used the centered log-ratio transformation with the interquartile log-ratio (IQLR) denominator and Welch's t-test, with the number of Monte Carlo Dirichlet instances set adaptively to dataset size [10]. Across all methods, incorporators were called at an adjusted p-value below 0.05 and an absolute log₂ fold change above 1. Because ALDEx2's IQLR denominator cannot construct its reference on highly sparse contrasts, we tested whether alternative denominators could recover these cases. Substituting the per-condition (zero) denominator allowed ALDEx2 to run on the failed contrasts and recovered additional true incorporators, but at a substantial cost in precision (evaluated against ground truth in our SIPSim benchmark; see Results), with false positives concentrated in density-resolved designs. We therefore retained the IQLR default [10].

For each taxon and differential abundance method, effect sizes across windows were combined using inverse-variance weighting (weights = 1 / max(padj, 0.01)), and window-level p-values were combined using Fisher’s method [10]. Directional consistency was quantified as the proportion of windows showing enrichment in the same direction, and method-level evidence was integrated using Stouffer’s Z-score method [11] to generate a single cross-method consensus p-value per taxon. Taxa were designated as isotope incorporators if they met all three criteria: adjusted p-value < 0.05, absolute log₂ fold change > 1, and directional agreement across windows > 0.5. Both fixed-window and sliding-window analyses were performed for all position- and density-resolved studies, but only sliding-window consensus results were incorporated into version 1 of SIPdb; all window- and method-level outputs were retained to preserve analytical provenance.

*Validation and Integration of Windowing Strategies for SIPdb version 1*

To validate these approaches, we applied both fixed- and sliding-window methods to all case 2 (*n* = 14) and case 3 (*n* = 4) datasets and compared their relative information loss or gain. For position-resolved studies without density information (case 2), the fixed-window approach grouped the heaviest 33% of fractions from isotopically labeled samples as the “heavy” window and compared them directly against the corresponding heaviest 33% of fractions from the natural abundance controls, while lighter fractions from both labeled and unlabeled gradients were excluded from the analysis, following the rationale of Youngblut et al., 2014. [12] When applying the sliding-window approach to position-resolved studies with few fractions (≤ 3), each fraction was analyzed as an individual window. For position-resolved gradients with ≥5 fractions, hierarchical clustering (Ward’s method with Euclidean distance) [8] was used to group adjacent positions (e.g., 2–3–4) into two to five windows. These groupings were applied only to contiguous gradient positions, preserving the physical ordering of fractions along the gradient. An alternative k-nearest-neighbor (KNN) [9] sampling approach was available for specific cases, adaptively selecting equal numbers of labeled and unlabeled samples (default: k=3 per group, adjusted based on total sample number) closest to each gradient position, maintaining statistical balance while capturing localized enrichment patterns. For density-resolved studies using the fixed-window method, windows were primarily focused on the heavy fraction (≥1.725 g/mL for stringent analysis, ≥1.700 g/mL for relaxed analysis), with optional borderline windows (1.710-1.725 g/mL) for intermediate incorporators. Sliding-window analyses for case 3 used the continuous density measurements directly and did not require clustering. Both windowing strategies were applied to all datasets to compare their performance and were included in the final version of the database.

Results from each window approach and each gradient case were compared within each gradient case to quantify how the choice of window strategy affected incorporator detection. For each taxon and method, effect sizes across windows were combined using inverse variance weighting [weights = 1/max × (*p*_adj_, 0.01)], and *p*-values were combined using Fisher's method. Direction consistency was assessed as the proportion of windows showing the same direction of effect. Cross-method consensus was established by combining method-level meta-*p*-values using Stouffer's Z-score method. Taxa were classified as incorporators if they exhibited consistent enrichment across windows (agreement score > 0.5), met statistical thresholds (*p*_adj_ <0.05), and showed substantial effect sizes (|log_2_FC| >1).

To determine which windowing strategy produced the most reliable incorporator calls and to create a unified, high-confidence output for SIPdb, we compared the fixed-window and sliding-window approaches within each gradient case. For every taxon, evidence across all windows was summarized using the four differential abundance methods implemented in our pipeline (DESeq2, edgeR, limma-voom, and ALDEx2). Window-specific effect sizes were combined using inverse-variance weighting, where weights were set to 1 divided by the maximum of the adjusted *p*-value and 0.01, and window-level *p*-values were combined using Fisher’s method. [13] Direction consistency was calculated as the proportion of windows in which the taxon showed enrichment in the same direction, and we required a consistency score greater than 0.5 for inclusion. Outputs from the four statistical methods were integrated using Stouffer’s Z-score approach to generate a single cross-method consensus *p*-value for each ASV. Within each method, results from multiple density windows were first consolidated into a method-level log_2_-fold change (log_2_FC) estimate and a consistency score describing window behavior. The consensus log_2_FC reported in SIPdb corresponds to the mean of these method-level log_2_FC values, while the agreement score represents the mean of the per-method consistency scores. Results from multiple differential abundance methods and windowing approaches were thus integrated to produce a single consensus incorporator decision per ASV per study and substrate, while retaining full method- and window-level provenance in the database. Both fixed-window and sliding-window analyses were performed for case 2 and case 3 datasets, but only the sliding-window summaries were used to compute the consensus statistics incorporated into version 1 of SIPdb. An ASV was designated a consensus incorporator when it simultaneously satisfied: agreement score > 0.5, consensus padj < 0.05, and log₂FC > 1. ASVs detected by only a single differential abundance method were retained in the consensus pipeline; in such cases, the consensus estimate reduces to the single method's result, ensuring that incorporators are not excluded in study designs where only one method was applicable. Because all three criteria must be met jointly, the consensus set represents a higher-confidence subset of the full union of individual methods. When users select the ‘consensus’ option in SIPdb, these consensus statistics are displayed alongside all underlying window records for transparency, resulting in multiple rows per ASV that share the same consensus values rather than representing independent consensus calculations.

*Validation with SIPSim synthetic data procedures*

Synthetic SIP benchmark datasets were generated using SIPSim (v0.3)[14] to evaluate the ability of the pipeline to recover taxa with known isotope-incorporation status. We generated 100 independent SIPSim simulations and converted each simulation into three analysis views: binary heavy/light, position-resolved multifraction, and density-resolved gradient datasets. Each simulation was constructed from genome-derived fragment GC distributions and synthetic community profiles drawn from a panel of 219 bacterial reference genomes (Information about genomes present in **Table** **S6**). Community richness varied across simulations, with 100, 150, or 219 taxa per synthetic community. The proportion of true isotope incorporators varied across simulations at 2%, 5%, 10%, 15%, 18% or 30% of taxa. Isotope incorporation intensity was varied using atom-percent enrichment levels of 25%, 50%, 75%, or 100%, which SIPSim converts into taxon-specific buoyant-density shifts according to the physical relationship between isotopic mass and DNA density. Parameter combinations were randomized across the 100 simulations rather than arranged as a complete factorial. Each simulation contained eight libraries: four natural-abundance control libraries and four isotope-labeled libraries (detailed information about each simulation in **Table S7**).

For each simulation, composition was generated with SIPSim communities, and per-genome fragment buoyant densities were modeled by generating 2,000 fragments per genome and building a fragment kernel density estimate (SIPSim fragment_KDE). Gradient diffusion and a diffusive boundary layer were then applied to the fragment KDE (SIPSim diffusion and SIPSim DBL) to reproduce the density dispersion characteristic of CsCl gradient fractionation. Isotope incorporation was applied only to designated incorporator taxa using SIPSim isotope_incorp with an explicit taxon-by-library incorporator map. We used a fixed-incorporator design for each simulation, with the same set of true incorporator taxa assigned to all labeled libraries, whereas control libraries were assigned zero isotope incorporation. SIPSim isotope incorporation was performed using an explicit taxon-by-library incorporator file, ensuring that designated incorporators were consistent across labeled replicate libraries. After simulation, ground-truth validation was performed using SIPSim buoyant-density shift summaries. A simulation was retained only when all designated incorporator taxa showed isotope-induced buoyant-density shifts in all four labeled libraries and no non-incorporator taxa showed isotope-induced shifts.

For each simulation, SIPSim generated fractionated count tables across CsCl gradients (SIPSim gradient_fractions), followed by simulated PCR amplification (SIPSim OTU_PCR) and sequencing-depth subsampling (SIPSim OTU_subsample). The resulting fraction-level count tables were converted into phyloseq objects in three formats: density-resolved datasets retained fraction-level buoyant-density values; position-resolved multifraction datasets retained gradient-position information, with position 1 representing the heaviest fraction; and binary datasets were generated by pooling fractions into heavy and light categories using buoyant-density thresholds. All the parameters used for the simulations are described in **Table S8**.

*Constructing the SIPdb relational database*

For the initial release of SIPdb, isotope incorporators were identified for each study dataset using the appropriate gradient case according to each study design. For position- and density-resolved studies, both fixed-window and sliding-window approaches were evaluated; however, only sliding-window consensus results were retained in SIPdb version 1 due to their greater stability across gradient configurations. Binary studies (case 1; n = 4) were analyzed as direct contrasts between heavy fractions of isotopically labeled and unlabeled samples. Position-based studies (case 2; n = 14) were analyzed using both fixed-window (pooling the heaviest 33% of fractions) and adaptive KNN sliding-window approaches. Density-resolved studies (case 3; n = 4) used the same dual windowing strategies, with windows defined by buoyant density ranges. All differential abundance analyses were performed on ASV counts, from which taxonomic incorporator designations were derived for extending cross-study comparability. An ASV was designated as an incorporator if it exhibited greater than 2-fold enrichment in the labeled nucleic acid pool (log_2_FC > 1) with Benjamini–Hochberg adjusted *p*-value (*p*_adj_) ≤ 0.05. We utilized dual density thresholds: the relaxed threshold (≥1.70 g/mL) to capture broader incorporation patterns with greater sensitivity, and a stringent threshold (≥1.725 g/mL) identifies high-confidence incorporators with reduced background. Both result sets are stored in the database (*sip results relaxed* and *sip results stringent* tables), allowing users to select thresholds appropriate for their research questions.

For each study, window, and substrate, differential abundance was evaluated using four complementary statistical frameworks. All method- and window-specific results were retained in SIPdb with full provenance, including window definitions and sample composition. Results from multiple differential abundance methods and windowing approaches were integrated to generate a single consensus incorporator call per ASV per study and substrate, as described above. For SIPdb v.1, KNN sliding windows adaptively selected k = 3 samples per group (labeled and unlabeled) nearest to each gradient position. For sliding-window analyses, window metadata included position ranges (*window start*, *window end*, *window center*), window mode (KNN or fixed), and sample sizes per group. Studies were excluded if they had insufficient sample sizes (<2 per condition), extreme sparsity (>98% zeros), or lacked proper controls. Quality metrics captured for each study included the total number of samples per condition (*n labeled*, *n unlabeled*), median library size, data sparsity (% zeros), and the number of ASVs passing filtering thresholds.

Results were compiled into a relational SQLite database. The primary results tables (*sip results relaxed* and *sip results stringent*) link differential abundance results to study metadata (*studies* table), taxonomy (*taxonomy* table), and sequences (*asv_sequences* table) through foreign keys. Each results row documents what was tested (*ASV identifier*), how it was tested (*analytical method*), the comparison performed (*analysis type, comparison type*), gradient information (*gradient position, density threshold*), and window context for sliding analyses (*window name, window mode, k per group*). The *asv sequences* table contains all representative V4 region sequences with associated sequence length, GC content, and full taxonomic lineage.

To facilitate data access and analysis, SIPdb provides several curated tables: *analysis view relaxed* and *analysis view stringent* combine differential abundance results with study, substrate, and taxonomy metadata into a single queryable table; *enriched_sequences* lists all significantly enriched ASVs (*p*_adj_ ≤ 0.05 and log_2_FC > 1) together with their sequences and taxonomy; and *consensus_both_thresholds* identifies ASVs classified as incorporators under both relaxed and stringent density thresholds, providing a high-confidence subset for cross-study comparisons. All representative ASVs were compiled into a BLAST nucleotide database to enable homology-based queries using BLAST+ (‘all_sequences_v1.fa’). [1] User-supplied 16S rRNA V4 sequences can be matched using standard BLASTn parameters, with incorporator status assigned based on sequence identity (≥97% identity for genus-level matches, or 100% identity over 251 bp for exact ASV-level matches).

Before finalizing version 1 of the SIPdb, we validated the following: (1) compared study-type detection accuracy (binary, multi-fraction, density classifications), (1) validated output completeness by confirming that the master table contained all required columns with expected data types and no missing critical values, (2) validated study coverage by verifying that all input datasets produced results in output files, and (3) validated file artifact integrity by checking that all expected output files (differential abundance tables, QC logs, summary reports, Excel workbooks) were generated without errors from all properly formatted phyloseq study inputs.

*Dashboard implementation and downstream analyses*

The dashboard comprises seven analysis modules. The *Incorporators Table* serves as the landing page, displaying ASVs meeting default enrichment criteria (*p*_adj_ < 0.05, log_2_FC > 1) with summary metrics (total incorporators, unique ASVs, studies represented), taxonomic search functionality, and exportable DOI-linked records. Five comparative analysis modules (*Environmental Analysis*, *Method Analysis*, *Isotopolog Analysis*, *Compare Isotopologs* and *Taxa Summary*) provide interactive visualizations including distribution bar charts with hover tooltips, summary statistics tables, and heatmaps of top 20 enriched taxa (by absolute log_2_FC), built with ggplot2, [15] plotly, [16] and viridisLite. [17] The *Environmental Analysis* module enables users to examine how isotope incorporation patterns vary across environments, identifying environment-specific incorporator guilds. The *Method Analysis* module enables users to compare how different analytical pipelines (e.g., edgeR, ALDEx2) influence incorporator detection and effect size estimates. The *Isotopolog Analysis* module enables users to explore how different labeled substrates structure incorporator communities and enrichment strength across taxonomic ranks. The *Compare Isotopologs* module enables side-by-side comparison of taxon enrichment between any two substrates at user-selected phylotype or taxonomic ranks (ASV through phylum), with ranked bar charts and exportable overlap tables showing taxa present in both datasets. The *Taxa Summary* presents all records for a chosen organism at any taxonomic rank, with distribution plots across environments and isotopologs annotated with log_2_FC values.

Finally, the dashboard has a BLAST Search module which accepts user-provided 16S rRNA gene sequences in FASTA format (single or multi-FASTA), supporting up to 30,000 sequences or file sizes ≤30 Mb. Sequences must overlap the V4 region (515F-806R primers) to yield results. Queries are executed with BLASTn against the SIPdb sequence database, returning tabular alignments linked to incorporator metadata (study, method, log_2_FC, p-value, environment, isotopolog, full taxonomy). Results are filterable by sequence identity threshold (default ≥97%) and incorporator status (enriched in heavy vs. light fractions based on log_2_FC sign). All filtered tables and figures are exportable (CSV format), DOI fields link directly to primary publications, and ASV identifiers are clickable to display full V4 sequences. The application uses a Shiny/SQLite backend with planned regular database updates as new SIP studies become available.

Downstream analyses using curated SIPdb outputs were performed as described in the Main Methods. Principal component analysis was conducted on scaled and centered variables using the prcomp function in R following log₁₀ transformation of count-based variables. Non-metric multidimensional scaling (NMDS) was performed on phylum-level incorporator relative abundances using Bray–Curtis dissimilarity with two dimensions and 100 random starts in the vegan package (v2.6-4) [18], and community differences were tested using PERMANOVA with 999 permutations. Re-analysis of non-SIP datasets followed the same processing and annotation procedures described above [19].

**REFERENCES**

1.	NCBI. *SRA Toolkit*. n.d. https://github.com/ncbi/sra-tools.

2.	Simpson A, Wood-Charlson EM, Smith M *et al.* MISIP: a data standard for the reuse and reproducibility of any stable isotope probing-derived nucleic acid sequence and experiment. *GigaScience* 2024;**13**:giae071. https://doi.org/10.1093/gigascience/giae071.

3.	Caporaso JG, Lauber CL, Walters WA *et al.* Global patterns of 16S rRNA diversity at a depth of millions of sequences per sample. *Proc Natl Acad Sci USA* 2011;**108**(supplement_1):4516–22. https://doi.org/10.1073/pnas.1000080107.

4.	Bolyen E, Rideout JR, Dillon MR *et al.* Reproducible, interactive, scalable and extensible microbiome data science using QIIME 2. *Nat Biotechnol* 2019;**37**(8):852–7. https://doi.org/10.1038/s41587-019-0209-9.

5.	Quast C, Pruesse E, Yilmaz P *et al.* The SILVA ribosomal RNA gene database project: improved data processing and web-based tools. *Nucleic Acids Research* 2012;**41**(D1):D590–6. https://doi.org/10.1093/nar/gks1219.

6.	The R Core Team. *R: A Language and Environment for Statistical Computing*, version 4.5.1. 2025.

7.	McMurdie PJ, Holmes S. phyloseq: An R Package for Reproducible Interactive Analysis and Graphics of Microbiome Census Data. *PLoS ONE* 2013;**8**(4):e61217. https://doi.org/10.1371/journal.pone.0061217.

8.	Youngblut ND, Buckley DH. Intra‐genomic variation in G + C content and its implications for DNA stable isotope probing. *Environ Microbiol Rep* 2014;**6**(6):767–75. https://doi.org/10.1111/1758-2229.12201.

9.	Ward JH. Hierarchical Grouping to Optimize an Objective Function. *Journal of the American Statistical Association* 1963;**58**(301):236–44. https://doi.org/10.1080/01621459.1963.10500845.

10.	Whitlock MC. Combining probability from independent tests: the weighted *Z* ‐method is superior to Fisher’s approach. *J of Evolutionary Biology* 2005;**18**(5):1368–73. https://doi.org/10.1111/j.1420-9101.2005.00917.x.

11.	*Statistical Methods for Meta-Analysis*. n.p.: Elsevier, 1985. https://doi.org/10.1016/C2009-0-03396-0.

12.	Youngblut ND, Barnett SE, Buckley DH. HTSSIP: An R package for analysis of high throughput sequencing data from nucleic acid stable isotope probing (SIP) experiments. *PLoS ONE* 2018;**13**(1):e0189616. https://doi.org/10.1371/journal.pone.0189616.

13.	Cover T, Hart P. Nearest neighbor pattern classification. *IEEE Trans Inform Theory* 1967;**13**(1):21–7. https://doi.org/10.1109/TIT.1967.1053964.

14.	Youngblut ND, Barnett SE, Buckley DH. SIPSim: A Modeling Toolkit to Predict Accuracy and Aid Design of DNA-SIP Experiments. *Front Microbiol* 2018;**9**:570. https://doi.org/10.3389/fmicb.2018.00570.

15.	Wickham H. *Ggplot2: Elegant Graphics for Data Analysis*, with Sievert C. Use R! Second edition, Cham: Springer international publishing, 2016.

16.	Sievert C. *Interactive Web-Based Data Visualization with R, Plotly, and Shiny*. Chapman and Hall/CRC the R Series. Boca Raton, FL: CRC Press, Taylor and Francis Group, 2020.

17.	Garnier S, Ross N, Antônio Pedro Camargo *et al.* *Sjmgarnier/viridisLite: viridisLite 0.4.0 (Pre-CRAN Release)*, version v0.4.0. Zenodo, 10 Apr. 2021. https://doi.org/10.5281/ZENODO.4678327.

18.	Oksanen J, Simpson GL, Blanchet FG *et al.* vegan: Community Ecology Package. 6 Sept. 2001, 2.7-2. https://doi.org/10.32614/CRAN.package.vegan.

19.	Li Z, Eshghdoostkhatami Z, Cupples AM. Biodegradation rates of low concentrations of 1,4-dioxane in impacted site sediments and agricultural soils and an investigation of the microorganisms and genes involved. *Environmental Pollution* 2025;**381**:126513. https://doi.org/10.1016/j.envpol.2025.126513.
